# Case Report: A Rare Condition of Abdominal Pain: Chemotherapy Induced Portal Vein Pneumatosis Mimicking the Bowel Necrosis

**DOI:** 10.3389/fsurg.2021.620908

**Published:** 2021-02-22

**Authors:** Chong-Cheng Chen, Yi Chen, Yue-Xin Zhang, Ze-Hua Chen, Kun Yang

**Affiliations:** ^1^Department of Nephrology, West China Hospital, Sichuan University, Chengdu, China; ^2^Department of Gastrointestinal Surgery, West China Hospital, Sichuan University, Chengdu, China

**Keywords:** portal vein pneumatosis, bowel necrosis, chemotherapy, surgery, abdominal pain

## Abstract

Portal vein pneumatosis is the presence of air in the portal venous system, which is one of the classic radiologic features of bowel ischemia or necrosis. However, there are several other morbidities that can have portal vein pneumatosis as a complication. This is a case of a 44-year-old man who suffered from severe abdominal pain after chemotherapy for soft tissue sarcoma of his left hip. The physical signs, laboratory findings, as well as the portal venous pneumatosis sign of the CT scan strongly indicated the probability of bowel necrosis and subjected the treatment decision of the patient finally to laparotomy. However, nothing abnormal except a segment of swollen small intestine was detected. Caution should be kept in mind when encountering a patient with suspected bowel necrosis following chemotherapy since several chemotherapeutic agents could cause portal vein pneumatosis. Diagnostic laparoscopy might be a better option for such cases.

## Introduction

Portal vein pneumatosis is the presence of air in the portal venous system, which usually presents along with pneumatosis intestinalis, and is regarded as one of the classic radiologic features of bowel ischemia or necrosis. Portal vein pneumatosis was first described in 1960, and the reported incidence of portal vein pneumatosis in necrotizing enterocolitis varied from 10 to 33% (1–3). Portal vein pneumatosis occurs commonly secondary to a series of diseases, including necrotizing enterocolitis, bowel ischemia, connective tissue disease, intestinal obstruction, inflammatory bowel disease, chronic obstructive pulmonary disease, trauma, colonoscopy, etc (4). Therefore, portal vein pneumatosis is not always associated with bowel necrosis or ischemia in all patients. This article discusses the rare occurrence of chemotherapy-induced portal vein pneumatosis mimicking bowel necrosis and highlights the lessons to be learned in order to avoid unnecessary surgery, particularly in the emergency setting.

## Case Presentation

**Figure 1:**
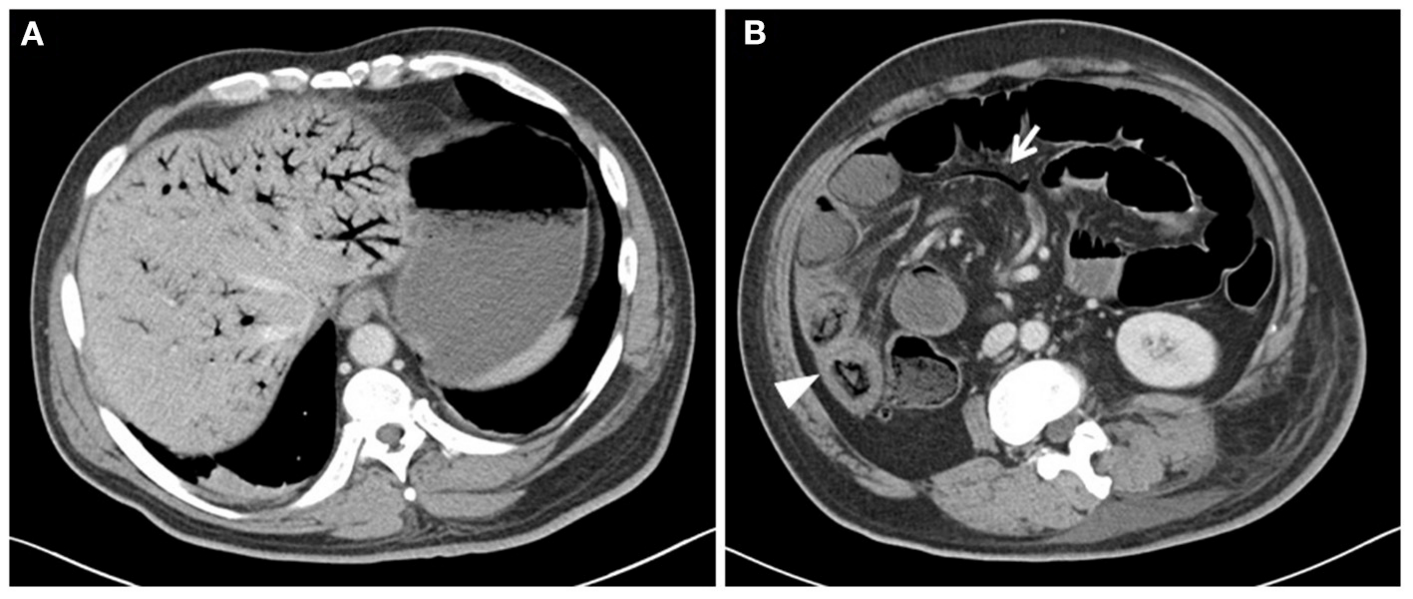
Abdominal computed tomography (CT) showed extensive portal **(A)**, mesenteric vessels gas [**(B)**, arrow], and dilated bowel loops. One segment of swollen small intestine [**(B)**, arrowhead] without obvious pneumatosis intestinalis was also observed in the CT scan.

A 44-year-old man was admitted for surgery due to soft tissue sarcoma of his left hip. Since the tumor was very large with a diameter of 30 cm and invaded the surrounding tissues, he had received the neoadjuvant chemotherapy (epirubicin 60 mg/m^2^ day 1, every 2 weeks) after the admission. Severe abdominal pain with diarrhea and vomiting were the complaints 3 days after the initiation of the second cycle of chemotherapy. There were no jaundice, abdominal distension, melena, and hematochezia. Physical examination revealed diffuse abdominal tenderness with mild rebound tenderness and guarding. Mild fever (37.6°C) and tachycardia (125 beats/min) were also demonstrated. Laboratory test results revealed remarkable neutrophilia (WBC 33.22 × 10^9^/L, Neutrophils: 98.4%) with normal bilirubin. The examination of stool showed no white blood cells. Computed tomographic images (CT) showed air in the portal (Figure 1A) and mesenteric vessels (Figure 1B, arrow) and dilated bowel loops. One part of edematous small intestine (Figure 1B, arrow head) without obvious pneumatosis intestinalis was also noticed. No thrombus was identified in the CT scan. As the differential diagnosis was intestinal necrosis, the necessity of surgery was told to the patient and his relatives. After obtaining their informed consent, an exploratory laparotomy was performed. However, nothing abnormal was detected, except for swollen small intestine from 100 to 150 cm distal to the Treitz ligament with a little ascites. No evidences of inflammation, including hyperemia of intestine and fibrous exudation of serosa, were detected on the segment of the swollen bowel, and its mesentery was normal with palpable pulses. The patient had a rapid recovery after operation, treated by antibiotics and parenteral nutrition. A follow-up CT scan showed complete resolution of portal vein pneumatosis 18 days after the exploratory laparotomy. Finally, the patient received left hemipelvectomy with the help of a temporary balloon occlusion of abdominal aorta successfully 20 days after the exploratory laparotomy. The post-operative pathological report showed that the tumor was high-grade sarcoma (unspecified/undifferentiation), and the immunohistochemical staining demonstrated PCK (+/±), EMA (–), eight factor (–), CD34 (–), CD31 (–), Fli-1 (–), INil (+), desmin (–), SMA (–), S-100 (–), and P53 (+) in the tumor cells. Three months later, the patient received interventional embolization of the branches of the bilateral internal iliac artery and radiotherapy to the tumor. However, the patient died from tumor progression 4 months later. During the period from exploratory laparotomy to his death, he had normal diet, and suffered no abdominal pain and symptoms of digestive tract. Table 1 shows the timeline with relevant data from the episode of care.

**Table 1:** The timeline with relevant data from the episode of care.

**Time**	**Event**
Day 0	Admission.
2 days later	The first cycle of neoadjuvant chemotherapy.
16 days later	The second cycle of neoadjuvant chemotherapy.
19 days later	Severe abdominal pain with diarrhea and vomiting were complained. An exploratory laparotomy was performed.
39 days later	The patient received left hemipelvectomy with help of a temporary balloon occlusion of abdominal aorta successfully.
128 days later	The patient received interventional embolization of branches of bilateral internal iliac artery and radiotherapy to the tumor.
240 days later	The patient died from tumor progression.

The patient's written informed consent was waived due to the retrospective nature, and the patient information was anonymized.

## Discussion

We reported the present case because of its rarity of the condition. Consequently, data collection of more cases is important and beneficial to improve the understanding of the features and risks of this unusual condition. Acquainting this uncommon situation for doctors may expedite the treatment of patients and avoid unnecessary interventions, especially in the context of emergency. Meanwhile, the treatment strategy of portal vein pneumatosis is challenging and needs to be discussed as well.

Portal vein pneumatosis is most commonly seen in conjunction with pneumatosis intestinalis. The possibility of the presence of portal vein pneumatosis increases with extensive degree of pneumatosis intestinalis (5). However, obvious pneumatosis intestinalis can be absent sometimes (6). The mechanism of portal vein pneumatosis is not clear. It may be a result of penetration of intraluminal gas through the bowel wall with impaired integrity, or it may be caused by invasion of gas-forming microorganisms into the portal venous system (6). Although portal vein pneumatosis is one of the classic radiologic features of bowel ischemia or necrosis, there are several other morbidities, such as inflammatory bowel disease, trauma, chemotherapy, etc., that can have portal vein pneumatosis as a complication (7). In the present case, no well-known etiological factors (such as ischemic or bacterial infectious enteritis, chronic obstructive pulmonary disease, mechanical intestine obstruction, or inflammatory bowel disease) were identified. Furthermore, the pattern of intraluminal air distribution was inconsistent with any vascular territory; therefore, an ischemic cause was not considered (4). In addition, no any other drug, which might lead to portal vein pneumatosis, was taken, no white blood cells were found in the stool, and no thrombus was identified in the CT scan. We therefore presumed that the portal vein pneumatosis may be caused by chemotherapy. Furthermore, diarrhea and vomiting as adverse effects of gastrointestinal toxicity associated with the administration of chemotherapy were observed that could strengthen our inference. Because the intestinal mucosa is highly proliferative, mucosal damage is often caused by chemotherapy, which subsequently influences the mucosal integrity of the digestive tract and results in portal vein pneumatosis (4). It has been reported that several chemotherapeutic agents, such as fluorouracil, vincristine, cisplatin, cyclophosphamide, paclitaxel, methotrexate, irinotecan, doxorubicin, and etoposide, were associated with portal vein pneumatosis (4). Our case suggested that epirubicin could also cause portal vein pneumatosis. Indeed, the overall incidence of portal vein pneumatosis has been calculated to be only 0.06–0.12%, by retrospectively reviewing radiologic results (8, 9). Since it was reported sporadically, however, there was no frequency regarding the portal vein pneumatosis after delivery of chemotherapy.

Several imaging modalities including abdominal plain radiographic imaging, ultrasonography, and CT could be used for the diagnosis and evaluation of portal vein pneumatosis, in which CT has been proven to be the most useful (10). Recently, frequent application of CT has led to an increased detection of portal vein pneumatosis. The CT features of portal vein pneumatosis usually show that the extension of branching lucencies could reach within 2 cm of the liver capsule, mainly in the anterior–superior aspect of the left lobe (11). However, intrahepatic portal vein pneumatosis should be differentiated from intrahepatic biliary dilatation and gas in the biliary tree (pneumobilia). In pneumobilia, the air tends to locate centrally and accumulate in the large bile ducts at the hilum (10). Portal vein pneumatosis is a non-specific finding seen from benign to life-threatening conditions. Considering the relatively high mortalities of patients with bowel ischemia or necrosis, compared with those without bowel ischemia, bowel ischemia or necrosis should be differentially diagnosed in patients with portal vein pneumatosis. Unfortunately, it is a challenge to determine whether the bowel ischemia or necrosis exists from CT scan sometimes. The clinical presentation and physical signs should be evaluated comprehensively, besides the results of the CT scan, to determine whether the patients have life-threatening ischemic condition requiring emergency operation or benign course that might be cured by conservative therapies.

Determining whether the patient would need surgical management or not is challenging since no definite parameter could confirm the diagnosis of bowel ischemia. Doctors have to balance the potential injury of unnecessary surgical intervention against the inevitable mortality if necrotic intestine is not resected. Therefore, the treatment decision depends on a combination of clinical findings, laboratory parameters, and radiological findings. Neither portal vein pneumatosis nor pneumatosis intestinalis independently can distinguish the transmural from partial thickness bowel ischemia, while the presence of both signs together highly indicates transmural bowel ischemia (91%) in patients with bowel ischemia (12). The necrotizing colitis should be suspected if new onset of signs of sepsis, shock, and bloody stool were found on the basis of abdominal distension and diarrhea (7). Portal vein pneumatosis and/or pneumatosis intestinalis along with the presence of any segment of swollen bowel with thickness >4 mm might portend the bowel necrosis (10). It has been reported that portal vein pneumatosis with acidosis and high lactate levels could harbinger the bowel necrosis (13). The appearance of free air in the abdominal cavity suggests the perforation of the intestine following the bowel necrosis, which is a widely accepted indication for operation. Patients with established bowel necrosis should be immediately operated, while clinically stable patients could be tentatively treated by conservative therapy (14), and conservative therapy includes intravenous fluid infusion, broad-spectrum antibiotics, close monitoring, and fasting. In the present case, the physical signs of fever, tachycardia, and abdominal tenderness with mild guarding, laboratory finding of increased white blood cells, as well as the results of CT scan strongly indicated the probability of bowel necrosis and directed the treatment decision to laparotomy. However, nothing abnormal was detected except swollen small intestine from 100 to 150 cm distal to the Treitz ligament with a little ascites. Therefore, caution should be kept in mind when encountering a patient with suspected bowel necrosis following chemotherapy. Diagnostic laparoscopy might be a better option for such cases, since exploratory laparoscopy could provide a less invasive approach, compared with open exploratory laparotomy. Minimally invasive techniques have been utilized to assess bowel viability in micropremmies with suspected necrotizing enterocolitis, and proven to be feasible and safe (15). Because bowel necrosis was highly suspected pre-operatively in the present case, and the operative field of view might be influenced and the possibility of iatrogenic injury to the intestine increased in laparoscopic surgery since dilatation and intraluminal gas accumulation of intestine were obvious in the CT scan, we chose open laparotomy to facilitate a planned intestine resection and anastomosis.

## Data Availability Statement

The original contributions generated in the study are included in the article/Supplementary Material, further inquiries can be directed to the corresponding author.

## Author Contributions

Y-XZ and Z-HC: acquisition, analysis, or interpretation of data for the work. C-CC and YC: drafting the work. KY: final approval of the version to be published. All authors contributed to the article and approved the submitted version.

## Conflict of Interest

The authors declare that the research was conducted in the absence of any commercial or financial relationships that could be construed as a potential conflict of interest.

## Abbreviations

CT: Computed tomography
PVP: Portal vein pneumatosis.
